# The Mediating Role of Callous–Unemotional Traits in the Relationship between Parental Aggression and Behavioral Problems among Chinese Preschoolers

**DOI:** 10.3390/children10061054

**Published:** 2023-06-13

**Authors:** Gengli Zhang, Yantong Zhu

**Affiliations:** 1 Faculty of Educational Science, Anhui Normal University, Wuhu 241000, China; 2 School of Comprehensive Human Science, University of Tsukuba, Tsukuba 305-8577, Japan

**Keywords:** parental aggression, behavioral problems, callous unemotional traits, Chinese preschoolers

## Abstract

It is well known that aggressive parenting is associated with behavioral problems among Western children in their early childhood, but this has rarely been examined among Chinese preschoolers. The purpose of this study is to explore the relationship between aggressive parenting, callous–unemotional traits (CU traits), and behavioral problems among a large Chinese preschool sample. Data were collected in Wuhu city, China, from 1879 preschoolers (54% of whom were male) with a mean age of 65.66 months (standard deviation = 9.41). Parents provided information about the frequency of aggressive parenting, children’s behaviors, and demographic characteristics via an online questionnaire. Mediation models were applied to analyze the associations between aggressive parenting, CU traits, and behavioral problems. Preschoolers’ age, gender, and family socioeconomic status were considered as covariates. The findings of our study revealed that higher frequencies of parental psychological and physical aggression were associated with high levels of CU traits, which were related to increased levels of preschoolers’ behavioral problems. This study extends previous studies by revealing a positive relationship between aggressive parenting and behavioral problems among Chinese preschoolers via CU traits and highlights the risks of aggressive parenting. Interventions for improving parenting strategies and lessening callous–unemotional traits should be developed to help reduce behavioral problems.

## 1. Introduction

Mental health problems affect more than 28% of Chinese children [1], and rates of impairment and persistence are found to be similar to those observed in children of older age [2]. Children with behavioral problems may have difficulties in a variety of areas, such as social interactions, troubled parent–child relationships, physical safety, expulsion from nurseries or preschools, delayed school readiness, academic issues, and health problems as adults [3,4,5,6]. Prospective longitudinal research supports the notion that antisocial behavior develops in the early years [7]. According to the ecosystem theory [8], the family is an important microsystem for preschoolers’ socialization, and some mechanisms in parent–child interactions and parenting practices (such as harsh or inconsistent discipline) contribute significantly to the development of these behavioral problems [9].

Parental aggression refers to a set of behaviors that can be harmful to the well-being of children and adolescents [10]. Psychological aggression is the use of psychological force with the objective of producing psychological pain or fear in children in order to punish or control their misbehavior [11,12]. Psychological violence against children includes yelling or screaming at them, as well as insulting and threatening them [13]. Slapping, hitting, spanking, and other physical acts of violence toward children are all examples of physical aggression; although these acts are not typically meant to cause harm to children, they can have lasting negative consequences [14]. Theories have emphasized that parental aggression may be associated with children’s behavioral problems. The cognitive theory suggested that children who experienced psychological aggression may form a cognition that they are bad children and thus more likely to exhibit problematic behaviors [15,16,17]. Social learning theory suggests that children may learn aggressive behaviors from their parents [18]. Attachment theory emphasizes that parental aggressions may lead to insecure attachment, which may cause later children’s behavioral problems [19]. Previous empirical studies have shown that aggression, both physical and mental, is harmful to children, especially in the early years when their brains are still being shaped [20]. A recent meta-analysis revealed that parental physical aggression has a negative effect on a child’s physical and social development and can result in mental health problems throughout the child’s lifespan [21]. Xing and colleagues found that prenatal physical aggression can be a predictor for externalized problems among Chinese elementary school children [22]. A 5-year longitudinal study also revealed that parental physical aggression is related to behavioral problems among elementary school children. Studies have also shown that parental psychological aggression may transmit intergenerationally and cause an increase in mental health problems in children [23]. However, few studies have explored the relationship between physical and psychological aggression and behavioral problems among Chinese preschoolers and the underlying mechanisms.

Callous–unemotional (CU) traits, which are characterized by a lack of empathy, prosociality, and guilt, are strongly associated with disruptive behavioral problems and cause substantial damage to children, families, and societies [24]. CU traits comprise one proposed pathway through which exposure to aggressive parenting is connected to child behavioral problems [25]. The adaptive calibration model proposes that CU traits develop as a coping strategy for dealing with stressful situations [26]. Children who experienced parental aggression tend to be accustomed to using CU traits to protect themselves against parental aggression, and CU traits are positively related to later behavioral problems [27]. According to attachment theory, children acquire a schema of close relationships that is provided via communication during their early childhood [28]. Low empathy and high CU traits may be a result of children who have experienced aggressive parenting, and these children develop unsafe attachments and have difficulty forming deep relationships [29]. According to Kerig et al. [30], children who grow up in dysfunctional homes may learn to deal with trauma by emotionally withdrawing, a strategy that may encourage the emergence of CU traits. The previous literature also robustly links CU traits to behavioral problems, such as behaviors that externalize and internalize problems, during early childhood [31,32,33]. Because CU traits are shown to be related to both aggressive parenting and behavioral problems, CU traits may act as a mediator between these two factors. Several studies also found a link between parental aggression and adverse results and suggested that CU traits may play a mediating role in this relationship. Wang and colleagues found that CU traits have a mediation role in the relationship between psychological aggression and moral disengagement among a Chinese college student sample [34]. Another study conducted among a Chinese male juvenile delinquent sample also found that CU traits mediated the relationship between parental aggression and offspring aggression [35]. In a Western sample, Carlson and colleagues [36] found that CU traits may mediate the role of parental psychological aggression and result in risk behaviors (i.e., engaging in violence).

To date, existing research has mainly focused on CU traits as a potential mediator in the relationship between aggressive parenting and behavioral problems in Western countries or examined middle-childhood participants, adolescents, or adults; few studies have examined this topic among children in their early childhood or those in an Eastern or Chinese context [37]. Studies have emphasized the importance of examining CU traits in early childhood [38]. CU behaviors emerge as early as ages 2–3 years old and can predict later CP [38]. Since research has shown that the foundations of CU behaviors, such as empathy [39], develop early in childhood [40], identifying effective factors that result in problematic effects and intervening early during childhood may be able to minimize the probability of children developing more severe types of CP later [41]. A significant concern is whether the larger context, notably parenting, influences the development of CU behavior, particularly in early childhood [38]. A previous study has shown that children in Hong Kong had higher CU trait scores (i.e., higher levels of “unemotional behavior”) than a group from the United States [42], which may be due to the Eastern cultural trait of suppressing emotional expression. According to Allen et al. [43], there are cultural disparities in the degree to which UK and Chinese schoolchildren endorse particular items on the Inventory of Callous–Unemotional Traits (ICU). Allen et al. [43] hypothesized that differences in item thresholds may have resulted from the phenomenon in which Chinese children consistently rate specific items substantially higher or lower than UK respondents due to societal norms, such as higher expectations of achievement among Chinese parents. Other researchers have proposed that further cultural distinctions between Eastern and Western societies, such as collectivism vs. individualism, may be responsible for the slightly altered risk processes and presentations of CU traits in Asian populations [44]. In collectivist East Asian societies, where a more substantial emphasis is placed on interpersonal connectivity and prioritizing the interests of the group/others over one’s own self-interest, CU traits may carry a higher degree of severity and impairment than in more individualistic Western nations [45].

Therefore, this study aimed to extend the current literature by examining the mediating role of CU traits in the relationship between aggressive parenting and behavioral problems among Chinese preschoolers. We developed the following hypotheses: (1) high frequencies of parental psychological aggression or physical aggression are correlated with high levels of preschool behavioral problems; (2) CU traits positively mediate the relationship between aggressive parenting and behavioral problems.

## 2. Materials and Methods

### 2.1. Sample and Procedure

Data were obtained in Wuhu, Anhui Province, China. Wuhu is in Central China and has a medium national economic status [46]. Between May and June 2022, eight kindergarten schools from seven districts, which included rural and urban areas in Wuhu City, were randomly selected in our study. The districts were chosen based on their socioeconomic status and the local density of children. Teachers and principals of kindergartens were made aware of the goals of the study. Prior to their children enrolling in kindergarten, parents were requested to provide information about their children’s health, such as any allergies, chronic illnesses, or ADHD. After the child was enrolled, the kindergarten arranged for a physical examination by a pediatrician to verify the child’s physical well-being. Children with health problems were not eligible for participation in the study. We also excluded from our study children whose parents suffered from physical or mental ailments or communication disorders. All parents of newly enrolled kindergarteners who met our inclusion criteria were extended an invitation to take part in our cohort study. Every parent gave informed consent after being fully briefed on the study’s goals, procedures, and their right to withdraw from the study at any time. The primary caregivers of the children received 1890 invitations, and 1879 of them gave their consent. Web-based questionnaires were distributed via the “WenJuanXing” online survey platform after all consent forms had been gathered. The online survey, which also included the factors under study and demographic data, was then distributed. Valid data were submitted by all 1879 caregivers, and these data were used for further study. The Anhui Normal University Ethics Committee gave its approval for this work.

The study included 1879 preschoolers (54% of whom were male) with a mean age of 65.66 months (standard deviation (SD) = 9.41). Mothers of preschoolers filled out the questionnaire the majority of the time (83.4%), followed by fathers (15.2%) and other family members (1.4%). In this study, 93.1% of the families’ annual income reached CNY 50,000, which indicates that the economic level of families was higher than the average Chinese family [46].

### 2.2. Measures

#### 2.2.1. Parental Aggression

Parental psychological aggression and physical aggression were assessed by the Chinese version of the Parent–Child Conflict Tactics Scale (CTSPC) [47], which has been widely used in China and has shown good reliability and validity [48]. Psychological aggression (4 items, i.e., shouted, yelled, or screamed at him/her) and physical aggression (13 items, i.e., spanked him/her on the bottom with your bare hand) subscales were used in this study. Parents reported how frequently they used specific behaviors toward their children in the previous six months, with 0 representing “never,” 1 representing “once,” 2 representing “twice,” 3 representing “3–5 times,” 4 representing “6–10 times,” 5 representing “11–20 times,” and 6 representing “more than 20 times.” We recorded numbers 3 to 6 as the midpoint of each category (i.e., 3 = 4 times, 4 = 8 times, 5 = 15 times, and 6 = 25 times) to obtain the frequency of aggressive behavior [47,49,50]. High scores indicated a high frequency of parental aggression. Cronbach’s alpha values in this study were 0.66 and 0.68 for psychological and physical aggression subscales, respectively.

#### 2.2.2. CU Traits

ICU-11 was used to measure preschoolers’ CU traits [51,52]. ICU comprises three subscales: callous (eight items, i.e., does not care who he/she hurts to get what he/she wants), unemotional (eight items, i.e., expresses his/her feelings openly), and uncaring (eight items, i.e., seems motivated to do his/her best in structured activities). The caregivers rated responses on a 4-point Likert scale ranging from 1 (not at all true) to 4 (definitely true). Higher scores indicated higher levels of CU traits. Wang et al. [52] tested the ICU-11 among Chinese children, and the results suggested that ICU-11 may be a promising assessment tool for research assessing callous–uncaring traits in Chinese children. In the Western preschool sample, researchers suggested that ICU-12 was suitable for preschoolers [53,54]. In the current study, we compared ICU-11 and ICU-12, and confirmatory factor analysis showed the better validity of ICU-11: comparative fit indices (CFI) = 0.984, Tucker–Lewis index (TLI) = 0.979, root mean square error of approximation (RMSEA)  =  0.053, and standardized root mean square residual (SRMR)  =  0.027. The Cronbach’s alpha of the ICU-11 in our sample was 0.82.

#### 2.2.3. Behavioral Problems

We applied the Strength and Difficulties Questionnaire (SDQ) in our study, which has been widely used in Chinese preschool children samples. This 25-item questionnaire includes 5 subscales: conduct problems (five items, i.e., often fights with other children), peer relationship problems (five items, i.e., picked on or bullied), emotional problems (five items, i.e., many worries), hyperactivity (five items, i.e., constantly fidgeting or squirming), and prosocial behavior (five items, i.e., helpful if someone is hurt) [55,56]. Primary caregivers rated their responses on a 3-point scale ranging from 0 (not true) to 2 (certainly true). The scores from the four subscales were summed, with total scores ranging from 0 to 50. A higher total score indicated a higher level of behavioral problems. Previous studies have shown the Chinese SDQ version can be used for Chinese preschool samples with good validity and reliability. The Cronbach’s α in our sample was 0.73.

#### 2.2.4. Covariates

Parents reported their children’s age, gender, and family socioeconomic status (SES) in the online questionnaire. We chose these as covariates based on previous studies [46].

### 2.3. Statistical Analysis

First, the mean, standard deviation, frequency, and percentage of all variables were determined using descriptive statistics. Second, correlation analysis was performed for the main variables. Then, Haye’s [57] PROCESS macro model 4 was used to examine the mediation model. Two separate models were developed to test the mediating role of CU traits in the relationship between aggressive parenting and preschooler behavioral problems. The 95% confidence intervals were estimated using 5000 bootstrap samples. In the data analysis, age, gender, and family SES were all considered as covariates.

## 3. Results

### 3.1. Descriptive Information

Table 1 provides descriptive statistics regarding this study’s participant and variable demographics. Scores of 15.41 ± 16.97 and 9.19 ± 13.86 were obtained for psychological and physical aggression, respectively. In terms of CU traits, the mean score was 15.41 ± 16.97. The mean total score for behavioral problems was 9.58 ± 4.26. Table 2 displays the bivariate relationships between the primary variables. There was a positive correlation between CU traits and behavioral problems, and these pertained to both psychological and physical parental aggresions There was also a positive correlation between CU traits and behavioral problems.

### 3.2. Mediation Analysis

Table 3 shows the association between preschoolers’ psychological aggression and problematic behavior, and this association is mediated by CU traits. The level of psychological aggression was directly related to behavioral problems (b = 0.03, *p* < 0.001). Positive associations were found between psychological aggression and CU traits (b = 0.05, *p* < 0.001) and between CU traits and behavioral problems (b = 0.45, *p* < 0.001). CU traits’ indirect effect was observed in the relationship between psychological aggression and behavioral problems (indirect effect = 0.01; Boot SE = 0.03; 95% CI = [0.01, 0.02]).

CU traits had a mediating effect on the relationship between physical aggression and preschoolers’ behavioral problems (Table 4). Physical aggression scores (b = 0.06, *p* < 0.001) displayed a significant positive direct influence on behavioral problems. The indirect effects of path models are presented in Table 4. Physical aggression was positively associated with CU traits (b = 0.06, *p* < 0.001), which in turn were associated with behavioral problems (b = 0.45, *p* < 0.001). We found that CU traits fully mediated the association between physical aggression (indirect effect = 0.02; Boot SE = 0.01; 95% CI = [0.01, 0.03]) and behavioral problems.

## 4. Discussion

Our findings indicate that phycological and physical aggression were significantly correlated with behavioral problems among Chinese preschoolers. The results further revealed the partial mediating role of CU traits in the relationship between aggressive parenting and behavioral problems.

The results support our first hypothesis that aggressive parenting is related to behavioral problems, which is consistent with previous studies. Empirical studies have shown that parental psychological and physical aggression are associated with children’s behavioral problems [37,58,59]. Theoretical perspectives may explain the association between psychological aggression and children’s behavioral problems. According to ecosystem theory [8], children’s behavioral problems were associated with their microsystem-level factors (i.e., parent–children relationship). Parental aggressions were considered a risk factor for parent–children relationships, which may cause children’s behavioral problems [60]. Rose and Abramson’s [15] cognitive theory also suggested that children who have experienced psychological aggression may believe that they are victims of psychological violence because they are bad children who will never be loved by anyone. Internalized [16] and externalized symptoms are associated with these types of cognitions [17]. Children’s behavioral problems are also frequently explained by social learning theory [18], which posits that children learn aggressive behaviors by modeling and imitating their parents’ physical punishment. Based on attachment theory, Steinberg [19] suggested that the key antecedents of child-onset delinquency are sentiments of rejection from the parent and having an insecure connection with parents. Children who frequently experienced aggressive parenting were more likely to have insecure attachment bonds with respect to their primary caregivers [61], which could contribute to later behavioral problems [62].

Additionally, we found evidence in support of our second hypothesis, which proposed that preschoolers’ CU traits act as a mediator between aggressive parenting and behavioral problems. According to the adaptive calibration model, CU traits may emerge as coping techniques in stressful environments [26]. Children who are exposed to aggressive parenting face substantial strain, and they often develop CU traits to defend themselves against aggressive parenting [63]. Therefore, children who have experienced more aggressive parenting are more likely to report CU traits [64]. For example, a child who is being psychologically assaulted may not react to the abuse, and this assists the child’s avoidance in admitting that there is abuse and prevents them from becoming cognizant of their upsetting emotions, keeping their relationship with the abuser intact. This interpretation is reinforced by research showing that emotional numbing acts as a mediator in the relationship between trauma and CU traits [27]. In contrast to previous studies conducted among a Chinese juvenile sample, in which physical aggression did not relate to CU traits, our study shows that exposure to physical aggression can increase CU traits. One explanation is that children who are exposed to physical aggression at a young age are comparatively more fearless to both punishment and the negative consequences of their behavior, increasing the risk of CU traits due to potentially separate etiological mechanisms [65]. Furthermore, CU traits have been recognized as an important construct for developmental models of psychopathology and have played a key role in expanding the idea of psychopathy to children and adolescents [66,67,68]. Empirical research has demonstrated that CU traits are a positive predictor of a variety of antisocial behaviors, including conduct issues, violence, and delinquency, in both clinical and community samples [67,69].

The main strength of this study is the utilization of a large preschool sample to explore the relationship between parental aggression and children’s behavioral problems. Few studies have examined the relationship between different aspects of parental aggression (e.g., psychological aggression and physical aggression) and behavioral problems among Chinese preschoolers, despite the fact that parental aggression has been thoroughly studied in Western samples. This may be due to the lack of a formal child protection system and cultural factors that prevent disclosure or acknowledgment [70]. Another strength of this study is that it reveals the mediating role of CU traits in the association between parental aggression and behavioral problems. There is a growing body of research focusing on CU traits in early childhood in Western countries; however, few studies explore the role of CU traits in Chinese preschool samples. Based on our results, interventions for CU traits can be designed in accordance with our findings in order to decrease the risk of behavioral problems in preschoolers who have experienced parental aggression.

The results of this study need to be interpreted within the context of some limitations. First, this study was cross-sectional, so causal relationships could not be determined, and applying cross-sectional mediation analyses may cause some bias in results; a longitudinal study may be better in achieving causal results [54]. Second, the genetic influence on CU traits and behavioral problems was not considered in this study; future studies should also consider genetic factors [71]. Third, bidirectional relationships between parenting, CU traits, and behavioral problems have been found in previous studies [72]. In the future, longitudinal repeated measures of parenting, CU traits, and behavioral problems should be used to test these bidirectional relationships. Fourth, this study used the midpoint of the CTSPC and may skew the results, and this may result in inaccurate findings. Fifth, although we found a significant indirect path, the magnitude of the weights (i.e., 0.1 and 0.2) suggests that these paths were not strong. We suggest that future studies should test more mechanisms underlying the relationship between parental aggression and behavioral problems. Furthermore, the generalizability of study results is geographically and ethnically/culturally limited because the study was conducted solely in China. Finally, all variables were reported by parents, which may result in shared method variance; we suggest that future studies should collect data from different responders (i.e., teachers, parents, and lab observers).

## 5. Conclusions

Behavioral problems, which can have long-term detrimental effects, are increasing among Chinese preschoolers. Therefore, the influencing factors and underlying mechanisms of behavioral problems warrant further exploration. To the best of our knowledge, this study is the first to reveal a positive association between aggressive parenting and behavioral problems among Chinese preschoolers via CU traits. Our results highlight the risks of aggressive parenting and the development of CU traits among Chinese preschoolers. Interventions should address parenting strategies and CU traits in order to help reduce behavioral problems among young children.

## Figures and Tables

**Table 1:** Demographic information of participants and descriptive information for all variables.

Variables	Category	*n* (%) or Mean ± SD
Child’s age (month)		65.66 ± 9.41
Gender	Boy Girl	1015 (54.0) 864 (46.0)
Father’s occupation	Unemployed, technical worker, or farmer Semi-technical worker or small business owner Technical worker or semi-professional Professional, officer, or owner of a mid-size business High-level professional or administrator	74 (3.9) 501 (26.7) 483 (25.7) 551 (29.3) 270 (14.4)
Mother’s occupation	Unemployed, technical worker, or farmer Semi-technical worker or small business owner Technical worker or semi-professional Professional, officer, or owner of a mid-size business High-level professional or administrator	394 (21.0) 341 (18.1) 481 (25.6) 562 (29.9) 101 (5.4)
Father’s education level	Primary school or below Middle school or below High school or vocational secondary school Vocational college degree Bachelor’s degree Master’s degree or above	13 (0.7) 241 (12.8) 329 (17.5) 451 (24.0) 741 (39.4) 104 (5.5)
Mother’s education level	Primary school or below Middle school or below High school or vocational secondary school Vocational college degree Bachelor’s degree Master’s degree or above	26 (1.4) 298 (15.9) 312 (16.6) 478 (25.4) 684 (36.4) 81 (4.3)
Family’s annual income	< CNY 50,000 CNY 50,001–10,0000 CNY 100,001–150,000 CNY 150,001–300,000 > CNY 300,000	130 (6.9) 401 (21.3) 566 (30.1) 591 (31.5) 191 (10.2)
Psychological aggression		15.41 ± 16.97
Physical aggression		9.19 ± 13.86
CU traits		18.34 ± 4.34
Behavioral problems		9.58 ± 4.26

**Table 2:** Bivariate correlations among the main variables.

	1	2	3	4	5	6	7
1. Age	--						
2. Gender	−0.024	--					
3. SES	0.051 *	−0.006	--				
4. Psychological aggression	−0.012	−0.068 **	−0.049 *	--			
5. Physical aggression	−0.027	−0.108 **	−0.082 **	0.591 **	--		
6. CU	−0.077 **	−0.079 **	−0.060 **	0.130 **	0.159 **	--	
7. BP	−0.027	−0.102 **	−0.111 **	0.270 **	0.265 **	0.494 **	--

Note: SES = socioeconomic status; CU = callous–unemotional traits; BP = behavioral problems. * *p* < 0.05; ** *p* < 0.01.

**Table 3:** Mediating effect of CU traits in the relationship between psychological aggression and preschoolers’ behavioral problems.

Predictors	Model 1 (Criterion CU)		Model 2 (Criterion BP)	
	b	t	b	t
CO: Age	−0.03	−3.28 **	−0.01	0.67
CO: Gender	−0.64	−3.21 **	−0.44	−2.66 **
CO: SES	−0.28	−2.24 *	−0.40	−3.82 ***
X: PA	0.03	5.32 ***	0.05	10.34 ***
ME: CU			0.45	23.37 ***
R^2^	0.03		0.30	
F	14.69 ***		157.03 ***	

Note: CO = covariates; X = independent variable; ME = mediating factor; SES = socioeconomic status; PA = psychological aggression; CU = callous–unemotional traits; BP = behavioral problems. * *p* < 0.05; ** *p* < 0.01; *** *p* < 0.001.

**Table 4:** Mediating effect of CU traits in the relationship between physical aggression and preschoolers’ behavioral problems.

Predictors	Model 1 (Criterion CU)		Model 2 (Criterion BP)	
	b	t	b	t
CO: Age	−0.03	−3.18 **	−0.01	0.79
CO: Gender	−0.57	−2.88 **	−0.39	−2.35 **
CO: SES	−0.25	−1.98 *	−0.38	−3.56 ***
X: PA	0.05	6.38 ***	0.06	9.12 ***
ME: CU			0.45	23.07 ***
R^2^	0.04		0.29	
F	17.84 ***		150.64 ***	

Note: CO = covariates; X = independent variable; ME = mediating factor; SES = socioeconomic status; PA = psychological aggression; CU = callous–unemotional traits; BP = behavioral problems. * *p* < 0.05; ** *p* < 0.01; *** *p* < 0.001.

## Data Availability

Not applicable.
